# Effects of Polyploidization on Morphology, Photosynthetic Parameters and Sucrose Metabolism in Lily

**DOI:** 10.3390/plants11162112

**Published:** 2022-08-14

**Authors:** Qian Zhang, Hao Hu, Yuzhou Jiang, Lianjuan Wang, Xiangfeng Kong, Yixuan Huang, Guixia Jia

**Affiliations:** 1 Beijing Key Laboratory of Ornamental Plants Germplasm Innovation & Molecular Breeding, National Engineering Research Center for Floriculture, Beijing Laboratory of Urban and Rural Ecological Environment, Key Laboratory of Genetics and Breeding in Forest Trees and Ornamental Plants of Ministry of Education, School of Landscape Architecture, Beijing Forestry University, Beijing 100083, China; 2 Ningbo City College of Vocational Technology, Ningbo 315100, China

**Keywords:** polyploidization, *Lilium*, photosynthesis, nonstructural carbohydrates (NSCs), sucrose metabolism

## Abstract

Polyploidization is widely used in ornamental plant breeding. The polyploids usually produce greater amounts of biomass. However, the alternations to sucrose metabolism that occur in lily during development after polyploidization induced using colchicine are poorly understood. In this study, compared with their allodiploid counterparts, allotetraploid lilies presented a larger total leaf area per plant and slightly delayed flowering time. Moreover, photosynthetic parameter measurements revealed a higher net photosynthetic rate (Pn), stomatal conductance (Gs), transpiration rate (Tr), and maximum Pn for allotetraploids than for allodiploids. Compared with allodiploids, allotetraploids also showed higher nonstructural carbohydrate (NSC) contents during development according to HILIC-CAD results. The expression levels of sucrose metabolism-related genes were higher in allotetraploids than in allodiploids at the same time points. The expression profiles of several target genes in allotetraploids were distinctly different from those in allodiploids. *Susy2*/*3* exhibited opposite expression profiles in allotetraploids and allodiploids, and the expression profiles of *SPS3* and *Susy2* were significantly correlated with sucrose content change trends in allodiploids and allotetraploids, respectively.

## 1. Introduction

Lily (*Lilium* spp.), an important floricultural crop species with great ornamental value, some with edible bulbs, occupies a considerable share of the market of commercially important plants [1]. Artificial polyploidy has been widely used as an important plant breeding method because of the superiority which polyploids often exhibit the “gigas” effect [2]. With the duplication of chromosomes in plants, agronomic traits, including morphology, flowering time, and stress resistance, have been shown to be altered after polyploidization [3,4,5]. A previous study in our laboratory showed that artificially induced polyploid lily presented significantly greater amounts of biomass, including longer and wider leaves, as well as slightly delayed flowering time compared to its allodiploid counterparts [6]. Polyploidization also often results in altered metabolism, especially in primary metabolites [7]. As pivotal substances in primary metabolism, sugars produced by photosynthesis provide nutrition and energy and are involved in plant development [8,9]. However, the alterations to sucrose metabolism during lily development after polyploidization remain unknown.

In the process of sucrose being translocated to sink organs, sucrose synthase (Sus) is responsible for sucrose catabolism and producing hexose under optimal conditions [10,11]. The molecular evidence shows that the sucrose degradation enzyme-related gene Susy is involved in leaf initiation and sink development, especially in crop species [12,13]. The Oriental lily hybrid *LohSusy* was found to take part in the sucrose cleavage and starch synthesis in the shoot-to-bulblet transition and displayed elevated expression [14].

Hexose can also be the resynthesis of sucrose by sucrose-phosphate synthase (SPS) and sucrose-phosphate phosphatase (SPP), which are two key enzymes that play a role in the synthesis of disaccharides [15,16]. The overexpression of SPS increased the leaf photosynthesis and flower numbers of transgenic tomato plants [17].

Sucrose is hydrolyzed into glucose and fructose [9]. Together with trehalose-6-phosphate (T6P), these sugars play signaling roles directly or through interactions with other signaling pathways involved in plant growth, developmental regulation, and stress responses [11,18,19]. T6P is a pivotal intermediate in the carbohydrate signaling modulation network that directly relays information about sugar status in plants [20,21]. T6P is produced from the metabolism of sucrose (hexose) catalyzed by trehalose-6-phosphate synthetase (TPS). Then, T6P is dephosphorylated by trehalose 6-phosphate phosphatase (TPP) to form trehalose [11,21].

The previous studies in our laboratory showed that relative to diploids, colchicine-induced allotetraploids have larger leaf area in *L.* × *formolongi* × Oriental hybrid [6]. To explore how allopolyploidization affects biomass via the sugar metabolism pathway during lily development, a new allopolyploidy hybrid, which was developed and selected by our laboratory that has yellow flowers and strong heat resistance and flowers in summer, was developed here on the basis of the crossing of *L*. × *formolongi* and an Asiatic hybrid. On the basis of the investigation of morphological traits and the growth rate of allodiploids and allotetraploids, a comparative analysis of photosynthesis-related physiology, nonstructural carbohydrate (NSC) contents, and expression profiles of sugar-metabolism-related genes in allodiploid and allotetraploid plants during development was performed in this study. The results could provide fundamental clues regarding how to increase biomass through the promoting effect of polyploidization on sugar metabolism in lily.

## 2. Results

### 2.1. Comparisons of the Morphology of Allotetraploids and Allodiploids

**Figure 1:**
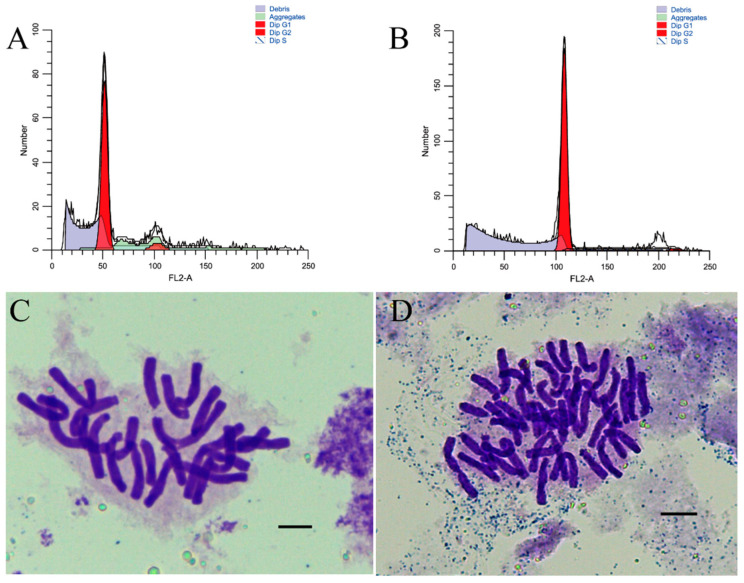
Ploidy confirmation of polyploidization−induced lily plantlets. (**A**,**B**) Flow cytometry histograms of the allodiploids (**A**) and allotetraploids (**B**). (**C**,**D**) Chromosomes in root tip cells from allodiploid (2n = 2x =24) (**C**) and allotetraploid (2n = 4x = 48) (**D**) plants; bars = 10 μm.

**Figure 2:**
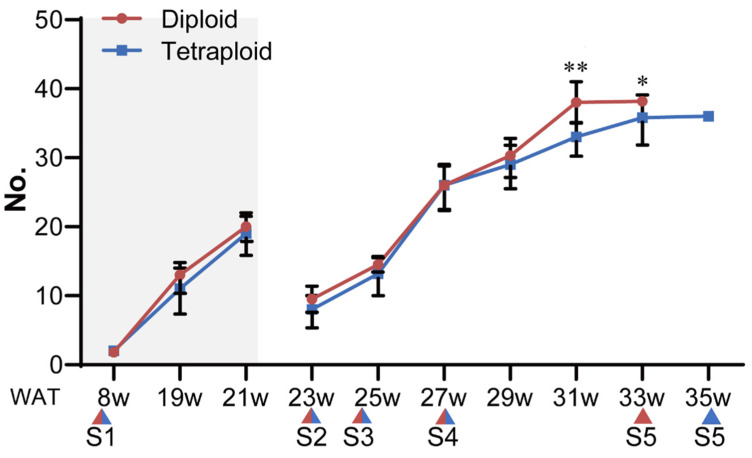
Development of allodiploid and allotetraploid lilies from transplanting until flowering. No. represents number of rosette leaves or internodes after bolting. The shaded bars in the chart represent the numbers of rosette leaves at the rosette−leaf stage. W represents weeks after transplanting. The triangles indicate the five different developmental stages (S1 to S5) of the allodiploids (red triangles) and allotetraploids (blue triangles). Significant differences between the samples of allodiploids and allotetraploids at the same time points were determined by Student’s *t*-tests (*, *p* < 0.05, **, *p* < 0.01). Error bars represent SDs (*n* = 10).

**Figure 3:**
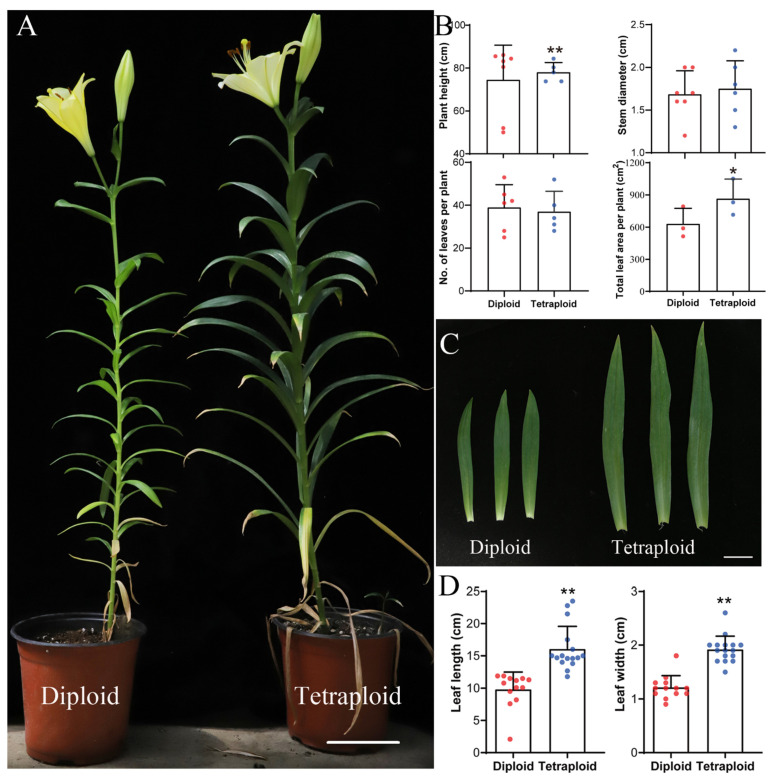
Morphological comparisons of allodiploid and allotetraploid lilies. (**A**) Allodiploids and allotetraploids at the flowering stage; bar = 5 cm. (**B**) Morphological comparisons of allodiploids and allotetraploids at the flowering stage. Error bars represent SDs (*n* = 5–7). (**C**) Leaves of allodiploids and allotetraploids; bar = 2.5 cm. (**D**) Length and width of leaves of allodiploids and allotetraploids. Error bars represent SDs (*n* = 11–15). Significant differences in plant height, leaf length, and leaf width between allodiploids and allotetraploids were determined by Student’s *t*-test (*, *p* < 0.05; **, *p* < 0.01).

On the basis of the results of flow cytometry (FCM) and chromosomal counts, we determined the ploidy levels of the induced plantlets (Figure 1). The allodiploids and allotetraploids were propagated in vitro and transplanted into a greenhouse. After the plantlets were transplanted, there was no significant difference in the vegetative growth rate between the allodiploids and allotetraploids (Figure 2). Moreover, the differences in morphological traits between allodiploids and allotetraploids were obvious (Figure 3). The average plant height, total leaf area, leaf length, and width of the allotetraploids were significantly greater than those of allodiploids, although the allotetraploids had less leaves. The average stem diameter per plant of adult allotetraploids was also higher than that of allodiploids (Figure 3B–D). Additionally, the leaves of the allotetraploid plants were darker green than those of the allodiploid plants, with a higher chlorophyll content and smaller Δa* value measured by spectrophotometer (Figure S1).

**Figure 4:**
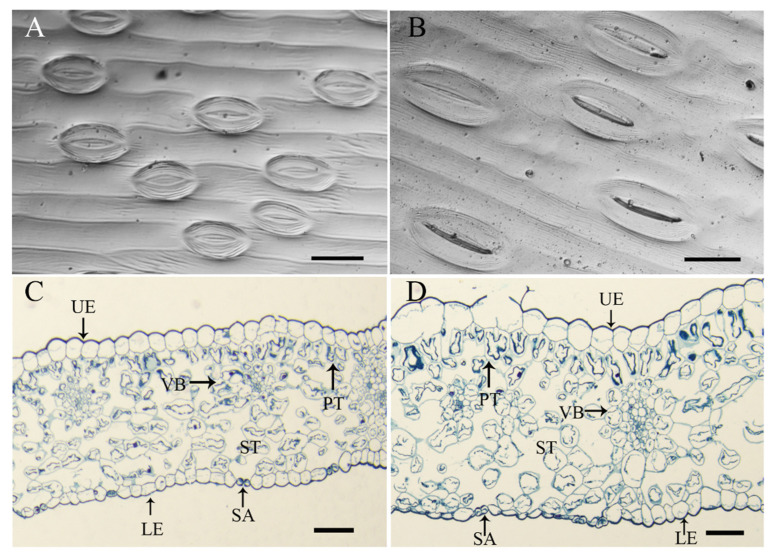
Stomata and anatomical structures of the leaves of allodiploid and allotetraploid lilies. Stomata within the leaf epidermis of allodiploids (**A**) and allotetraploids (**B**); bar = 50 μm. Leaf anatomical structures of allodiploids (**C**) and allotetraploids (**D**). UE, upper epidermis; LE, lower epidermis; PT, palisade tissue; ST, spongy tissue; VB, vascular bundle; SA, stoma; bar =100 μm.

The stomatal traits of the leaves of the allodiploid and allotetraploid plants were determined. The stomatal length and width of the leaves significantly increased after polyploidization (Figure 4, Table 1). However, the stomatal density of the allotetraploid plants was significantly lower compared with that of the allodiploid plants.

The leaf anatomical structure was also affected by polyploidization. Compared with that of the allodiploid plants, the leaf thickness of the allotetraploid plants was significantly greater because of the greater thickness of the upper epidermis, palisade tissues, spongy tissues, and vascular bundles (Figure 4, Table 2). Among these structures in the allotetraploid plants, the spongy tissue was 74.24% thicker and contributed the most to the significant increase in leaf thickness compared with that of the allodiploid plants.

### 2.2. Changes in Photosynthetic Parameters

**Figure 5:**
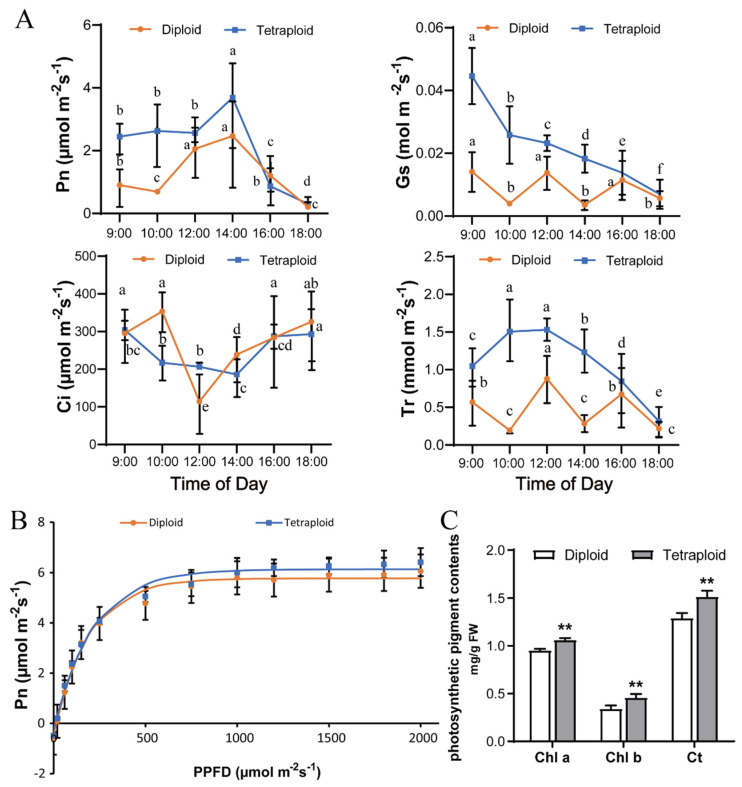
Photosynthetic parameters of the hybrid allotetraploid lily and its allodiploid counterpart. (**A**) Diurnal variation in photosynthetic parameters, including the net photosynthetic rate (Pn), stomatal conductance (Gs), transpiration rate value (Tr), and intercellular carbon dioxide concentration (Ci), in allodiploid and allotetraploid plants. The values represent the means ± SDs of at least three individuals of each ploidy type. The letters indicate significant differences in photosynthetic parameter values among the samples of allodiploid and allotetraploid plants according to one−way ANOVA with post hoc Dunnett’s test, respectively (*p* < 0.05). (**B**) Light−response curves of the photosynthetic rate of allodiploids and allotetraploids. PPFD: photosynthetic photon flux density; each icon in the figure represents the average Pn. The values represent the means ± SDs of at least three individuals of each ploidy type. (**C**) Photosynthetic pigment contents in allodiploids and allotetraploids. Chl a, chlorophyll a; Chl b, chlorophyll b; Ct, total chlorophyll. The data are the means ± SDs (*n* = 3–5). Significant differences in the photosynthetic pigment contents between the allotetraploids and allodiploids were determined by Student’s *t*-test (**, *p* < 0.01).

The photosynthetic parameters of allodiploids and allotetraploids were compared. The constructed curves of diurnal variations of photosynthesis revealed that the net photosynthetic rate (Pn), stomatal conductance (Gs), and transpiration rate value (Tr) values of allotetraploid plants were almost always higher than those of allodiploids at similar time points within a given day, although they displayed different diurnal variation tendencies (Figure 5A). The change trends of the diurnal variation in these photosynthetic indicators shifted after polyploid treatment. The Pn peaked at 14:00 and subsequently tended to decrease in both allodiploid and allotetraploid plants. It increased from 10:00 to 12:00 in the allodiploids, whereas it remained high and stable in the allotetraploids during the same period. Gs continued to decrease from 9:00 in the allotetraploids. However, the value tended to fluctuate in the allodiploids. A similar change trend was obtained for the Tr curve of the allodiploids. The Tr of the allotetraploids increased from 9:00–10:00 but then gradually declined. In contrast, the intercellular carbon dioxide concentration (Ci) of the allotetraploids was lower than that of the allodiploids at similar time points except at 12:00. The lowest Ci was recorded at 12:00 for the allodiploids and at 14:00 for the allotetraploids.

The light response curve demonstrated that the Pn per leaf area of the allotetraploids was higher than that of the allodiploids under the same saturating light conditions (Figure 5B). Our analysis of the light response curve also confirmed the result and indicated that the photosynthetic capacity (maximum photosynthetic rate (Pmax)) of the allotetraploids (6.13 µmol m^−2^s^−1^) was also higher than the Pmax of the allodiploids (5.78 µmol m^−2^s^−1^) (Table S2). Both the light saturation point (LSP) and the rate of dark respiration (Rd) of the allotetraploids were also higher than those of the allodiploids. However, there was no difference in light compensation point (LCP) or apparent quantum yield (AQY) between the allodiploids and allotetraploids.

The photosynthetic pigment contents in the allodiploid and allotetraploid plants were also measured and compared in this study. The chlorophyll a (Chl a) and chlorophyll b (Chl b) and total chlorophyll contents in the allotetraploids were significantly higher than those in the allodiploids (Figure 5C).

### 2.3. Increased NSC Contents in Allotetraploids

**Figure 6:**
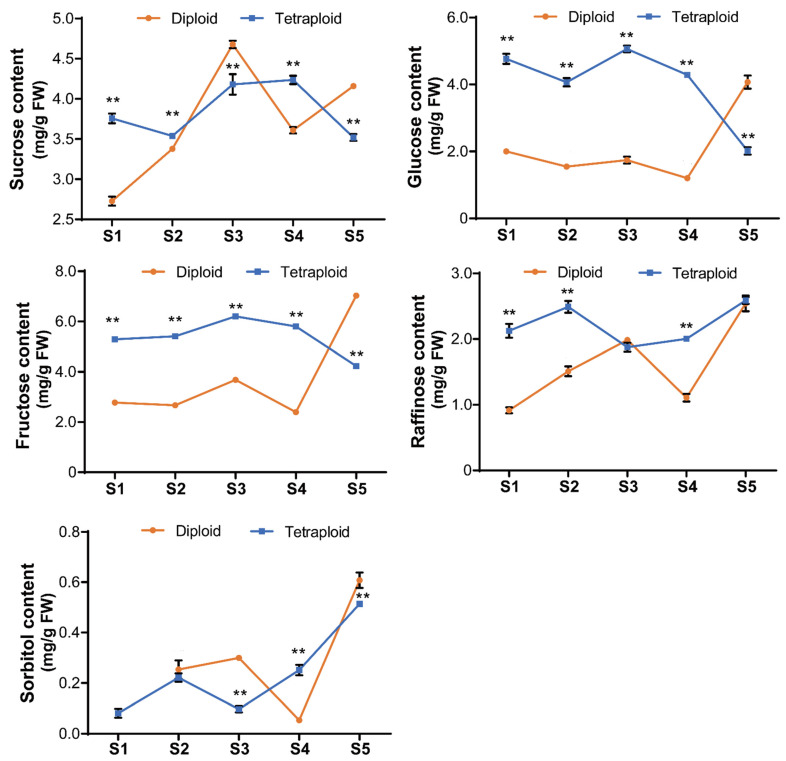
Nonstructural carbohydrate (NSC) contents in allodiploid and allotetraploid lilies during development. S1–S5 represent the five developmental stages. Significant differences between allodiploids and allotetraploids were determined by Student’s *t*-test in allodiploids and allotetraploids, respectively (**, *p* < 0.01). The data are the means ± SDs of three biological replicates with three technical replicates.

To explain the increased biomass after polyploidy treatment, we measured the NSC contents in allodiploid and allotetraploid plants from the juvenile vegetative stage to the visible flowering bud stage. The results showed that the glucose, fructose, and raffinose contents in the allotetraploids were higher than those in the allodiploids at the same time points before the visible floral bud stage (Figure 6). In both the allodiploids and allotetraploids, the glucose content and the fructose content exhibited similar change trends during lily development and flowering. However, in the allodiploids, the glucose content gradually decreased until S4, and a marked increase occurred after that stage. Similarly, after increasing from S1 to S3 and decreasing from S3 to S4 in the allodiploids, the fructose content significantly increased and ultimately peaked at S5. In the allotetraploids, both the glucose content and the fructose content peaked at S3 and gradually declined before flowering. However, the sucrose content in the allodiploids was higher than that in the allotetraploids at S3 and S5. The sucrose content in the allodiploids also continued to increase from S1 to S3 and fluctuated in the subsequent stages. In the allotetraploids, the sucrose content displayed a change trend similar to that of the glucose and fructose contents and reached its highest value at the S4 stage. Additionally, in the allodiploids, the change trend of the raffinose and sorbitol contents was similar to that of the sucrose content. In the allotetraploids, the raffinose and sorbitol contents increased from S1 to S2 and from S3 to S5.

### 2.4. Expression Patterns of Sucrose-Metabolism-Related Genes

**Figure 7:**
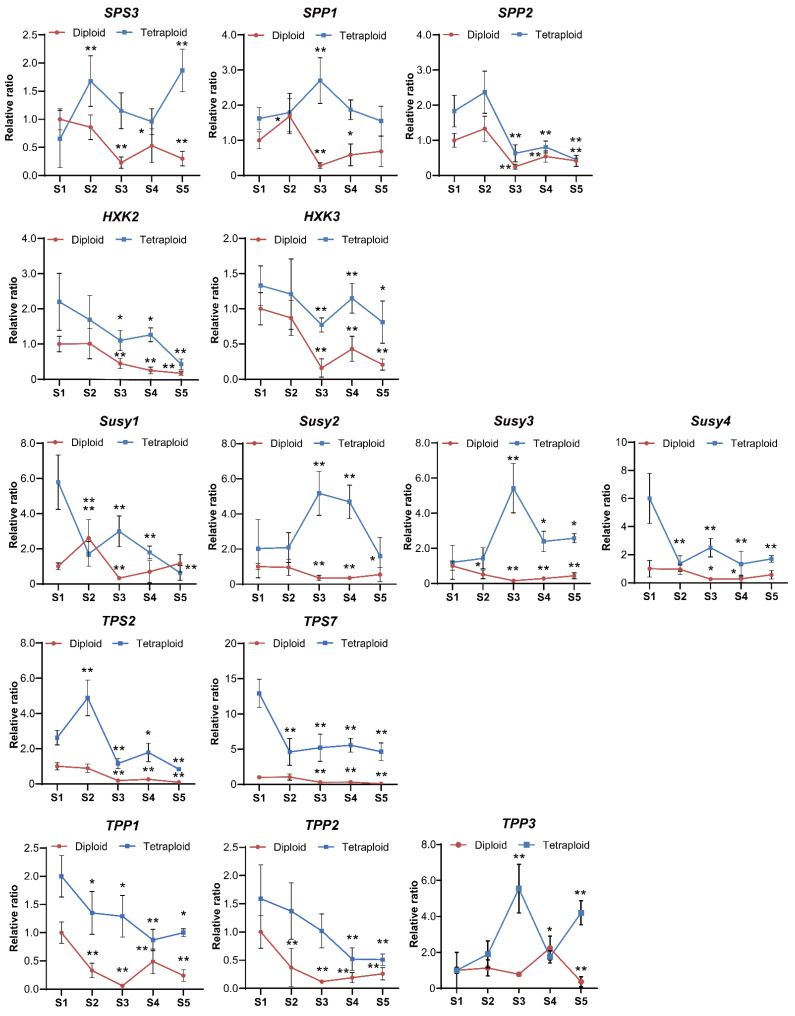
Expression patterns of sucrose-metabolism-related genes in allodiploid and allotetraploid lilies during development and flowering. S1–S5 represent the five developmental stages. The normalized expression levels in the allodiploids at S1 were arbitrarily set to 1. The data are the means ± SDs of three biological replicates, with three technical replicates. Significant differences between all the samples and plantlets of allodiploids and allotetraploids at S1 were determined by Student’s *t*-test (*, *p* < 0.05, **, *p* < 0.01).

To explore the change in sucrose metabolism at the molecular level after chromosome duplication in lily, the expression patterns of sucrose-metabolism-related genes were monitored in allodiploids and allotetraploids from the juvenile vegetative stage to the visible bud stage. Remarkably, the results showed that the expression levels of most tested genes in the allotetraploids were higher than those in the allodiploids at the same time points during development (Figure 7).

**Figure 8:**
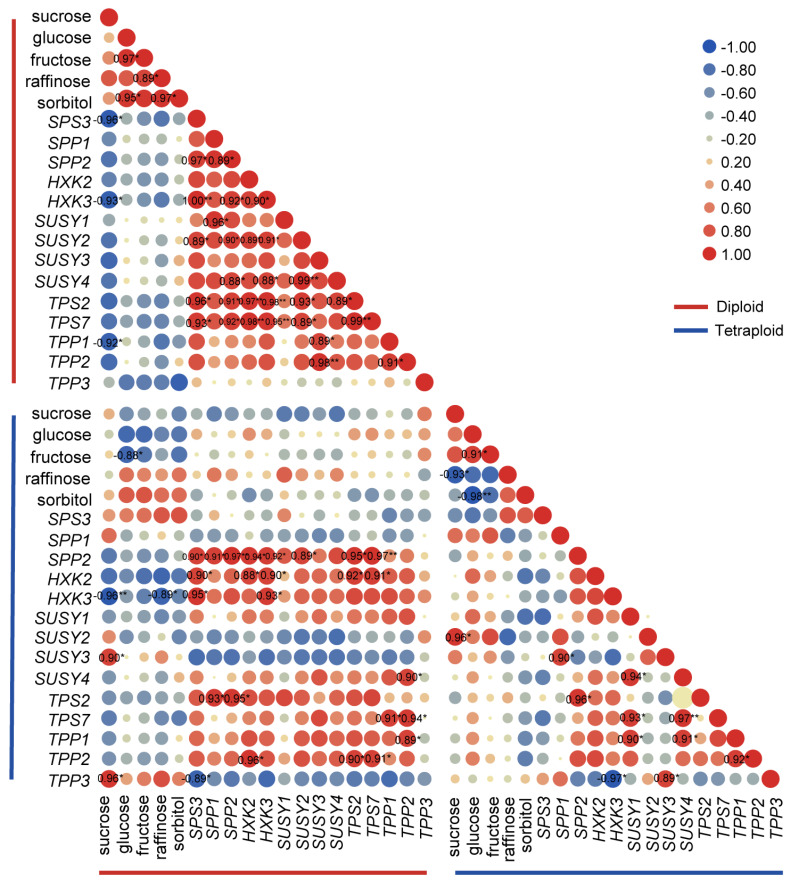
Correlation between two variables, nonstructural carbohydrate (NSC) contents and the expression level of sugar−related genes, in allodiploids and allotetraploids during development. Colors of blue to red represent levels of correlation. The numbers in circles are Pearson correlation values that are significantly different at *p* < 0.05 (*) or *p* < 0.01 (**).

**Figure 9:**
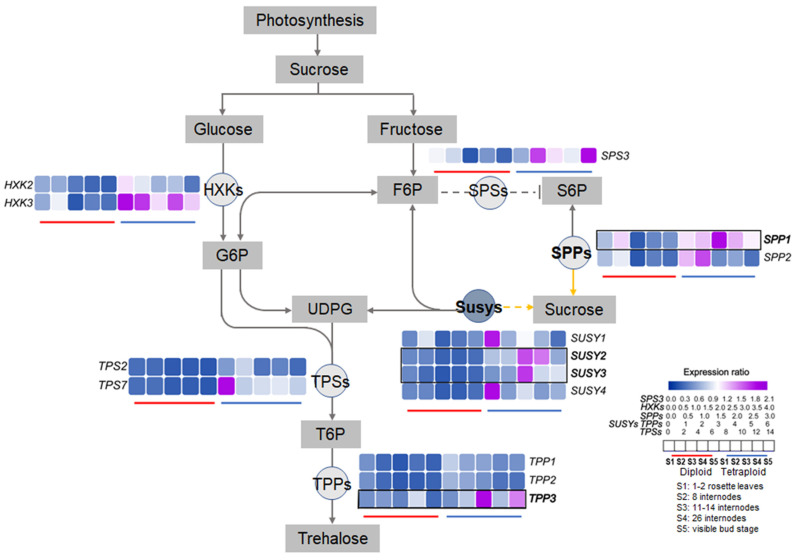
Putative sucrose metabolism−mediated regulatory network in lily hybrid allodiploids and their allotetraploid counterparts during development, with sorghum taken as a reference [22]. UDPG, uridine diphosphate glucose. G6P, glucose 6−phosphate. F6P, fructose 6−phosphate. The arrows of the dotted lines represent the potential regulatory relationships between two genes or metabolites. The grey and yellow connection lines with arrows represent sucrose cleavage and synthesis processes, respectively. The black−framed portion represents the expression levels of genes that displayed opposite expression patterns during lily development after polyploidization.

There were two different types of expression profiles for the tested genes during development. The expression profiles of several gene family members, such as *SPP2*, *HXK2*/*3*, and *TPS2*, showed nearly the same change trends during lily development after polyploidization (Figure 8 and Figure 9). Our correlation analysis also showed similar results that there were significant positive correlations between the expression levels of *SPP2*, *HXK2*, and *HXK3* in the allodiploids and allotetraploids (Figure 8). The other tested genes, such as *Susy1*, *Susy2*, and *Susy3*, displayed altered or opposite expression patterns during lily development after polyploidization. Furthermore, there was a significant positive correlation between fructose content and glucose content in both the allodiploids and the allotetraploids (Figure 8).

In the allodiploids, for the genes whose expression patterns were altered, the expression levels of *SPS3*, *Susy2*/*3*/*4*, and *TPP1*/*2*/*3* decreased from S1–S3 but then increased at subsequent stages. The expression levels of *SPP1*, *Susy1*, and *TPS2*/*7* peaked at S2 but then fluctuated before the visible bud stage. In the allotetraploids, more diverse expression profiles of the tested genes were observed. First, similar to those in the allodiploids, *SPS3*, *SPP1*, *Susy2*/*3*, *TPS2*, and *TPP3* exhibited the highest expression levels at S3 or S2. Among these genes, *SPS3*, *Susy3*, and *TPP3* also displayed increased expression levels before flowering. Second, the expression levels of *Susy1*/*4* and *TPS7* decreased from S1 to S2 and from S3 to S5. Additionally, the expression of *TPP1*/*2* decreased continuously until S4.

The results of the correlation analysis showed that the expression profiles of *SPS3*, *SPP1*, *Susy1*, and *TPP3* between allodiploids and allotetraploids were negatively correlated to different degrees (Figure 8). Furthermore, there were negative correlations between the expression levels and NSC contents in the allodiploids and allotetraploids during development (Figure 8). For example, the sucrose content and expression levels of *SPS3*, *HXK3*, and *TPP1* were significantly negatively correlated in the allodiploids. Furthermore, in the allotetraploids, the sucrose content was significantly positively correlated with the expression level of *SUSY2*.

## 3. Discussion

To improve characteristics and restore fertility, chromosome doubling is employed in the plant breeding process. Moreover, DNA content has been shown to be correlated with cell surface and cell size [23,24]. Thus, polyploidization resulted in various altered plant morphological characteristics, such as increased leaf size, color intensification, and stem diameter, in this study. These results were consistent with common alterations observed for other ornamental cultivars or crop species, such as orchid (*Dendrobium nobile* Lindl.) and energy willow (*Salix viminalis* var. Energo) [4,25]. In addition, polyploid formation is accompanied by structural and biochemical changes, including genomic restructuring, gene loss, and changes in gene expression.

Photosynthetic capacity is the primary physiological factor associated with dry matter production and the growth of polyploids [26]. Previous studies have reported distinct differences in photosynthesis after polyploidization, and most polyploids exhibit improved photosynthetic characteristics, including increased chlorophyll contents and photosynthetic capacity [27,28,29]. In tetraploid pak choi (*Brassica rapa* ssp. *chinensis*), mulberry (*Morus alba* L.), and *Liriodendron sino-americanum*, the increased photosynthetic capacity increases their biomass relative to allodiploids [27,30,31]. In the present study, the photosynthesis was improved after polyploidization, and the photosynthetic model also changed with chromosome duplication according to the distinct diurnal variation tendencies of the Pn, Gs, Tr, and Ci values between allodiploids and allotetraploids, which was different from the results of previous studies on barley [3], FO lily hybrids [28], *L. rosthornii*, *L. primulinum* var. *orchraceun*, and *L. regale* [32]. Therefore, we suggest that the increased photosynthetic efficiency and altered photosynthesis led to more energy and carbon for cell division and expansion in allotetraploids, which contributes to their increased leaf area and more biomass.

Sugars, including sucrose and its cleavage hexose (glucose), have been shown to primarily regulate cell proliferation and expansion as signals [33,34]. In this study, we suggest that the increased NSC contents contributed to larger cell size such as in palisade tissues, spongy tissues, and vascular bundles, in leaves through the sugar signaling pathway. Additionally, compared to allodiploids, larger leaves and stems were shown in allotetraploids that did not show a significant retardation of development. These increased NSC contents may provide more substrates to other anabolic pathways and produce more energy to defend against genomic stress on cells following chromosome doubling during the vegetative growth in lily.

The differences in the expression of sugar-metabolism-related genes between allodiploids and allotetraploids during development may also be closely correlated with the increased NSC contents and leaf area after polyploidization. The higher expression levels of most tested genes in allotetraploids compared with allodiploids may be due to elevated gene contents. Several sugar-metabolism-related genes exhibited distinctly different expression patterns between the allodiploids and allotetraploids. These results suggest that the functions of these genes whose expression patterns were altered after polyploidization may also contribute to the altered morphological and physiological traits of allotetraploids. In contrast, the tested genes that had similar expression profiles during plantlet development, regardless of ploidy level, such as *SPP2* and *HXK2*/*3*, might be core components and play conserved central roles in sugar signaling during lily development.

Among the sugar-metabolism-related genes whose expression patterns were altered following polyploidization, *SPP1*, which is involved in sucrose synthesis, displayed opposite expression patterns in the leaves of allodiploids and allotetraploids at the same time points, indicating that *SPP1* plays different roles in the development of lily after polyploidy treatment. Additionally, studies on the positive relationship or opposite effect between SPS activity and sucrose concentration have been reported for different sugarcane lines [10,35,36]. In this study, the expression patterns of *SPS3* also showed a negative relationship with the change trends of sucrose in lily, indicating the repressive role of this gene in sucrose synthesis in lily. However, considering the posttranscriptional regulation of SPS activity [36], the role of *SPS* in sucrose metabolism and lily growth still needs to be further investigated. Furthermore, the expression patterns of Sus-encoding gene *Susy2* displayed negative correlations with the change trends of sucrose contents in allodiploids. However, these expression patterns showed positive correlations in allotetraploids suggesting that Susy isoforms play different roles in the sucrose metabolism pathway during lily development as a result of polyploidization. Additionally, previous study has proven that overexpression of *Sus* promotes leaf development and expansion in transgenic potato plants probably through sugar signaling [12]. Similarly, in this study, *Susy2/3* exhibited significantly increased expression from S2 to S4 in allotetraploids, indicating *Susy2*/*3* may involve in the leaf expansion and internodes extension in allotetraploids after bolting. However, *Susy 2*/*3* as well as *Susy1* showed opposite expression trends in allodiploids. Whether it is the opposite expression trend of *Susy2*/*3* result in the increased leaf area after polyploidization through sugar signaling pathway need to be further studied.

The expression of TPS- and TPP-coding genes is modulated by sugars [37]. In Arabidopsis, sucrose can marginally induce *AtTPS1* expression [38]. Moreover, in this study, the expression profiles of *TPS2* and *TPS7* as well as sucrose content change trends were negatively correlated in allodiploids and allotetraploids, respectively, during development. Thus, we hypothesize that sucrose represses the expression of *TPS2*/*7* in lily allodiploids and allotetraploids. Furthermore, *TPS1* plays an important role in flowering initiation in Arabidopsis [39]. *LfTPS1*/*3*/*5* display high expression levels at the lily vegetative juvenile stage [40]. Similarly, high expression levels of *TPS2* and *TPS7* at S1 and S2 occurred in allodiploids and allotetraploids in this study, indicating that *TPS2*/*7* play roles during vegetative growth. However, the functions of these genes in the flowering of allodiploids and allotetraploids need to be further studied.

A previous study in our laboratory confirmed that morphology could be an indicator of the vegetative juvenile stage and the adult development stage in lily. Bolting indicates that seedlings have entered the flowering induction stage [41]. In this study, in addition to *TPS2*/*7*, most tested genes exhibited the highest or lowest expression levels at S2 or S3 when the plantlets had internodes. These sugar-metabolism-related genes that showed remarkably altered expression levels at the rosette-leaf stage or after bolting may therefore play pivotal roles in lily flowering induction. Their functions in flowering regulation are the main focus of our future research.

## 4. Materials and Methods

### 4.1. Plant Materials

Scales from bulbs of FA lily hybrids, which were produced by a cross between *L*. × *formolongi* ‘Raizan 3’ (2n = 2x = 24) and the Asiatic hybrid, were used to generate polyploid plants using colchicine (0.05% (w/v)) in vitro. After the induction of chromosome polyploidization, the ploidy levels of the induced plantlets were determined by chromosomal counts and FCM using young root tips and leaves following previously established protocols [6,42,43].

Bulbs with the same diameters (approximately 1.3 cm) produced by allodiploids and allotetraploids, which were derived via tissue culture, were transplanted and cultivated in a greenhouse (Shun Yi district, Beijing, China; 116°15′51′′ N, 40°33′32′′ E) in August. Fresh leaves from the middle of at least three plants at five different developmental stages, i.e., S1 (2–3 rosette leaf stage), S2 (8–9 internode stage), S3 (12–14 internode stage), S4 (25–26 internode stage), and S5 (1 cm visible floral bud stage), were collected and stored at −80 °C for further NSC content analysis and sucrose-metabolism-related gene expression analysis (Figure 1E). All samples consisted at least three independent biological replicates, and all experiments consisted of three repetitions.

### 4.2. Phenotypic Characteristic and Growth Evaluations

After transplanting, the growth of the plantlets, including the rosette leaves, internode numbers, and time until emergence of visible buds, was monitored during development and flowering. The morphological traits of adult allodiploid and allotetraploid plants, including their height and total leaf areas per plant, were measured for the plants with visible floral buds (1 cm). At the same time, the stem diameter, length, and width of leaves were investigated from the base and middle of these plants. The leaf area was calculated on the basis of scans of the leaves on grid paper using Adobe Photoshop CS5 software (Adobe Systems, Incorporated, San Jose, CA, USA). Determination of the leaf color was performed using a spectrophotometer (Nippon Denshoku NF555, Tokyo, Japan) according to the instruction manual.

### 4.3. Leaf Anatomical Structure and Stomatal Characteristics Observations

Leaf segments (5 × 5 cm) of mature and completely expanded leaves from the middle of allodiploid and allotetraploid plants with approximately 22 internodes were fixed in a solution of FAA (38% formalin/acetic acid/50% ethanol = 5:5:90; v/v/v) and prepared for paraffin sectioning. The paraffin sections were processed according to a previous protocol, with slight modifications [28,44]. After dehydration using graded ethanol solutions (70%, 95%, 100%) and being cleaned using xylene, the samples were embedded in paraffin. The paraffin blocks were cut into 10 μm sections by a rotary microtome (Leica RM2245, Heerbrugg, Switzerland) and stained with fast green and safranin solutions. The sections were subsequently observed under a microscope (Leica DM500, Heerhrugg, Switzerland). For each sample, at least three slices were imaged with the LAS V4.4 system. The whole-leaf thickness, epidermis, palisade, and spongy mesophyll tissues and vascular bundles in each sample were randomly measured by Adobe Photoshop CS5 software (Adobe Systems, Incorporated, San Jose, CA, USA).

Similarly, fresh, mature leaves of adult allodiploid and allotetraploid plants were collected for stomatal characteristic analyses. The stomatal characteristics were analyzed using the nail polish impression method according to previous methods [32,45]. At least three slices of each sample were imaged with the LAS V4.4 system, and the stomatal length, width, and density were recorded and analyzed. A stomatal index was obtained by dividing the stomatal length by the stomatal width.

### 4.4. Photosynthetic Pigment and Photosynthetic Parameter Measurement

Fresh leaves from the middle of allodiploid and allotetraploid plants with 22 internodes were collected for the extraction of photosynthetic pigments according to previous methods [39,40,41,42,43,44,45,46]. The leaves were submerged in a 96% (v/v) ethanol solution in the dark at room temperature for approximately 24 h. The Chl a and Chl b contents in the allodiploid and allotetraploid plants were obtained on the basis of their absorbance at 470, 649, and 665 nm determined by the use of a spectrophotometer (Thermo Fisher Scientific, Shanghai, China) according to the methods of a previous study [47].

Photosynthetic parameters including the Pn per leaf area, Gs, Ci, and Tr of allodiploid and allotetraploid plants were measured using an LI-6400XT portable photosynthesis system (LI-COR, Lincoln, NE, USA) according to the manufacturer’s instructions (https://www.licor.com/env/support/LI-6400/manuals.html (accessed on 10 February 2022)). The diurnal variations in photosynthesis were measured every two hours from 9:00 to 18:00 under natural light.

Data used for plotting the light response curve were collected between 8:00 and 12:00 on sunny days according to the methods of a previous study [28]. After acclimation under saturating light conditions of 2000 μmolm^−2^ s^−1^ for 20 min, the photosynthetic parameters were measured by the use of a graded photosynthetic photon flux density (PPFP) series in which the intensity was decreased from 2000 to 0 μmolm^−2^ s^−1^. Light response curves for each type of plant were subsequently plotted using Photosynthesis Assistant software version 1.1 (Dundee Scientific, Dundee, UK). Thus, we determined the LSP, LCP, and Pmax from the curves.

### 4.5. NSC Content Measurements

The contents of NSCs, including sucrose, glucose, fructose, and raffinose, in the leaves of allodiploid and allotetraploid plants at five developmental stages during development and flowering were measured using the hydrophilic interaction liquid-chromatography-charged aerosol detection (HILIC-CAD) method according to a previous study, with modifications [48]. Two milliliters of 80% acetonitrile solution was added to 0.5 g of ground sample, after which the mixture was fully shaken. Then, ultrasound was conducted in a low-temperature water bath for 30 min using a PS40A ultrasonic cleaner (Shen Hua Tai, Shen Zhen, China). After centrifugation at 4 °C at 12,000 × *g* for 5 min, the supernatant was collected. The supernatants from the two repeated procedures were collected and brought to a volume of 5 mL. One milliliter of supernatant was filtered and used to measure the NSC contents using an Ultimate 3000 HPLC (Thermo, Germering, Germany) coupled with a Corona CAD Ultra instrument (Thermo, Chelmsford, MA, USA). The column was a Waters XBridge Amide type (250 mm × 4.6 mm, 3.5 µm).

### 4.6. Expression Profile Analyses of Sucrose-Related Genes

Total RNA was extracted from the leaves of allodiploid and allotetraploid plants via an EASYspin Plus Plant RNA Kit (Aidlab Biotechnology Co., Ltd., Beijing, China). Reverse transcription was performed using a ReverTra Ace qPCR RT Master Mix together with gDNA Remover (Toyobo, Shanghai, China) to synthesize cDNA. Homologous sequences of NSC-related genes were obtained from the transcriptomes of *L*. × *formolongi* (Razan No. 2) and the Asiatic hybrid ‘Annemarie’s Dream’ in our laboratory (data not published). The sequences of the primers used for genes amplification in the allodiploid and allotetraploid plants are presented in Table S1. RT-qPCR was performed using THUNDERBIRD SYBR qPCR Mix without Rox (Toyobo, Shanghai, China) according to a previously published protocol [49]. *Actin* was used as reference gene for normalizing the gene expression data [50].

### 4.7. Data Analysis

Statistical analyses and Pearson’s correlation analysis were performed using SPSS Statistics 17 software (IBM, Inc., Armonk, NY, USA). Figures were constructed using GraphPad Prism 5 and Adobe Photoshop CS5 software.

## 5. Conclusions

The effect of polyploidization on lily development through sugar metabolism was investigated in this study. The development of an allodiploid lily hybrid and its allotetraploid counterpart was measured and compared. Most sucrose-metabolism-related genes presented higher expression levels and altered expression patterns after polyploidization, and some of them were correlated with sucrose content change trends during development. The opposite expression change of sugar-metabolism-related gene *Susy2* may result in increased leaf area after polyploidization through the signaling pathway. These results could provide an improved understanding of the mechanism underlying sugar metabolism and important polyploidization for use in ornamental plant breeding.

## Figures and Tables

**Table 1:** The stomatal size and density of allodiploid and allotetraploid *Lilium* FA hybrids.

Ploidy	Stomatal Length (μm)	Stomatal Width (μm)	Stomatal Length-Width Ratio	Stomatal Density (/mm^2^)
Allodiploid	82.16 ± 5.87b	28.10 ± 1.35b	2.92a	77.96b
Allotetraploid	129.83 ± 6.12a	43.85 ± 1.35a	2.96a	23.86a
Difference (%)	58.02	56.04	1.37	69.39

The values represent the means ± standard deviations (SDs) of at least three individuals of each ploidy type. The letters indicate significant differences in leaf stomatal size and density between allodiploid and allotetraploid plants according to Student’s *t*-test (*p* < 0.05). *n* = 3–5.

**Table 2:** Leaf anatomical structure of allodiploid and allotetraploid *Lilium* FA hybrids.

Ploidy Level	Leaf Thickness (µm)	Upper Epidermis (µm)	Lower Epidermis (µm)	Palisade Tissue (µm)	Spongy Tissue (µm)	Vascular Bundle (µm)
Allodiploid	403.34 ± 100.96b	57.16 ± 17.06fg	40.56 ± 15.98i	57.77 ± 10.59d	31.40 ± 13.92gh	29.85 ± 6.81hi
Allotetraploid	601.30 ± 32.27a	64.33 ± 15.84f	47.12 ± 12.73gh	72.68 ± 15.39c	54.71 ± 17.89e	37.53 ± 11.04fg
Difference (%)	49.08	12.54	16.17	25.81	74.24	25.73

The values represent the means ± standard deviations (SDs) of at least three individuals of each ploidy type. The letters indicate significant differences from leaf anatomical structure of allodiploid and allotetraploid plants according to one-way ANOVA with post hoc Dunnett’s test (*p* < 0.05).

## Data Availability

Not applicable.

## Supplementary Materials

The following supporting information can be downloaded at https://www.mdpi.com/article/10.3390/plants11162112/s1, Figure S1: The value of the leaf color difference in diploids and tetraploids. Table S1: Specific qRT-PCR primers for sugar-metabolism-related genes in diploids and tetraploids. Table S2. Leaf photosynthetic parameters associated with diploid and tetraploid *Lilium* FA hybrids.
