# Supplementary material for: Effects of Polyploidization on Morphology, Photosynthetic Parameters and Sucrose Metabolism in Lily
Source: Plants (Basel). 2022 Aug 14;11(16):2112. doi: 10.3390/plants11162112 (PMC9413479; doi:10.3390/plants11162112)
Supplement: Supplementary file 1 [file plants-11-02112-s001.zip › Supplementary Figure.pdf]

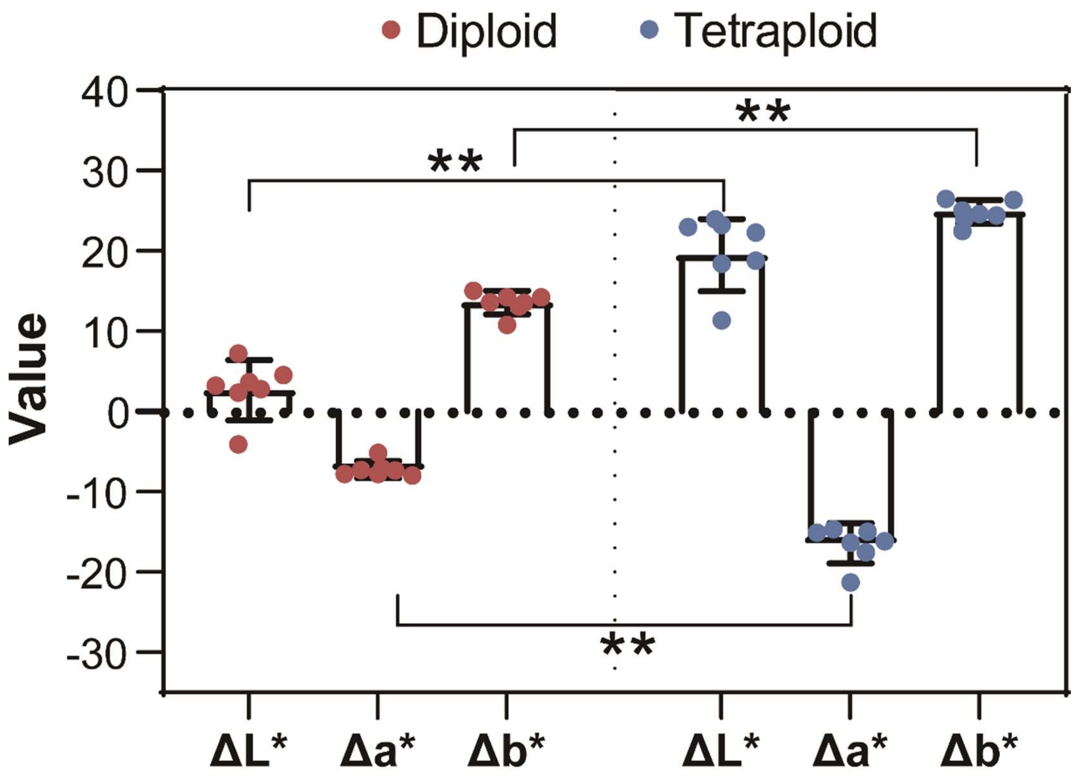

Figure S1. The value of the leaf color difference in diploids and tetraploids. The values represent the means ± SDs of at least three individuals of each ploidy type. Significant differences in the value between the tetraploids and diploids were determined by Student's *t* tests (**, $p<0.01$). △L* represents the difference in brightness between the samples and reference standards. △a* represents the difference in red/green between the samples and reference standards, where △a*- represents green. △b* represents the difference in yellow/blue between the samples and reference standards.
